# Comparing virtual consults to traditional consults using an electronic health record: an observational case–control study

**DOI:** 10.1186/1472-6947-12-65

**Published:** 2012-07-08

**Authors:** Ted E Palen, David Price, Susan Shetterly, Kristin B Wallace

**Affiliations:** 1 Department of Internal Medicine, Colorado Permanente Medical Group, Institute for Health Research, Kaiser Permanente, Denver, CO, USA; 2 Department of Education, Colorado Permanente Medical Group, Institute for Health Research, Department of Family Medicine, University of Colorado Denver Health Sciences Center, Denver, CO, USA; 3 Institute for Health Research, Kaiser Health Plan Colorado, Denver, CO, USA; 4 Knowledge Management and Implementation, Support, Kaiser Health Plan Colorado, Denver, CO, USA

**Keywords:** Consultations, Referrals, Electronic medical record, Value of information, Physician satisfaction, Patient satisfaction

## Abstract

**Background:**

Patients have typically received health care through face-to-face encounters. However, expansion of electronic communication and electronic health records (EHRs) provide alternative means for patient and physicians to interact. Electronic consultations may complement regular healthcare by providing “better, faster, cheaper” processes for diagnosing, treating, and monitoring health conditions. Virtual consultation between physicians may provide a method of streamlining care, potentially saving patients the time and expense of added visits. The purpose of this study was to compare physician usage and patient satisfaction with virtual consultations (VCs) with traditional consultations (TCs) facilitated within an EHR.

**Methods:**

We conducted an observational case–control survey study within Kaiser Permanente, Colorado. A sample of patients who had VCs requested by physicians (N = 270) were matched with patients who had TCs requested by physicians (N = 270), by patient age, gender, reason for the consult, and specialty department. These patients (VC and TC), were invited to participate in a satisfaction survey. In addition, 205 primary care physicians who submitted a VC or TC were surveyed.

**Results:**

During the study period, 58,146 VC or TC were requested (TC = 96.3%). Patients who completed a satisfaction survey (267 out of 540 patients, 49.4% response rate) indicated they were satisfied with their care, irrespective of the kind of consult (mean 10-point Likert score of 8.5). 88 of 205 primary care physicians surveyed (42.9%) returned at least one survey; VC and TC survey response rates and consulted departments were comparable (p = 0.13). More TCs than VCs requested transfer of patient care (p = 0.03), assistance with diagnosis (p = 0.04) or initiating treatment (p =0.04). Within 3 weeks of the consultation request, 72.1% of respondents reported receiving information from VCs, compared with 33.9% of the TCs (p < 0.001). Utility of information provided by consultants and satisfaction with consultations did not differ between VCs and TCs.

**Conclusions:**

Referring physicians received information from consultants more quickly from VCs compared with TCs, but the value and application of information from both types of consultations were similar. VCs may decrease the need for face-to-face specialty encounters without a decrease in the patient’s perception of care.

## Background

Traditionally, patients receive health care primarily by face-to-face encounters. Some authors suggest that 25% to 70% of all patients seeking care do not need a face-to-face appointment with a physician [1,2]. “Virtual medicine” using telecommunication and computerized means is being studied in various venues in efforts to provide coordinated care [3-5]. Virtual outreach may reduce the need for face-to-face appointments, reduce unneeded contacts with the health care system, increase patient satisfaction and improve patient health status. Secure, internet-based consultations may complement regular healthcare by providing “better, faster, cheaper” processes for diagnosing, treating, and monitoring health conditions [6-8]. Virtual consults may allow clinicians to receive guidance without added patient cost and inconvenience of physically going to the specialist, and may improve access to sub-specialists by “freeing up” some appointments for use by other patients needing in-person visits (unpublished, Price DW, 2009). Physicians have on average up to two questions about patient care per patient encounter [9-12], but search for answers less than half the time [9,10,12,13], with only a 50-75% success rate in finding answers [10,12,14-18]. Electronic “virtual” consultations may help clinicians obtain answers. However, even with increasing availability of electronic communication, less than 5% of consultations occur by email [14]. In at least one study, electronic communication between family medicine physicians and specialists was described as an efficient tool [19]. Electronic communication and increasing use of electronic health records (EHRs) make virtual consultation between physicians a promising method of streamlining care, potentially saving patients the time and expense of added visits. Younger generations of patients, who increasingly use electronic communication and social networking may come to expect virtual models of healthcare [20].

Shershneva and colleagues described a model of how physicians learn from specialty consultations [21]. Earlier pilot work [22] suggests that answers obtained from consultations have the potential to affect care that physicians provide for subsequent patients. Other than this work, to our knowledge, the extent to which information provided during physician-to-physician consultation is used in the care of the index patient or subsequent patients has not been well described. A few studies have investigated virtual consultation using the electronic messaging capabilities of an EHR system within an integrated healthcare environment [23-25]. Researchers have evaluated the use of telemedicine for remote consultations [26-29]. A recent meta-analysis showed a consistent pattern of improved primary care–specialist collaboration when interactive communication methods were employed [30]. A recent demonstration project showed how the use of an EHR has the potential to improve coordination of care between members of a patient’s care team but using an EHR to facilitate inter-office communication is still a barrier [31]. Detailed descriptions are lacking which examine the use of an EHR with an embedded secure messaging system between primary care and specialty care physicians in the context of an integrated health care delivery system.

We conducted an observational study comparing primary care referring physician perceptions on the value of virtual consultations (VCs) compared to traditional consultations (TCs). We quantified and classified the types of VCs and TCs requested, by whom, and for what purpose. We also surveyed patients who had either type of consult to gauge their impression of the care they received. We hypothesized that there would be no difference in physician satisfaction with the consultation process, the immediate clinical utility of the information from the consultant, the use of received information in subsequent patients, and overall satisfaction with the consultation by the physician or by the patient. We also hypothesized that referring physicians would receive information from a VC sooner than from a TC.

## Methods

### Study setting, design, and population

Kaiser Permanente Colorado (KPCO), a large group model integrated delivery system, provides healthcare for a diverse population of over 500,000 members in the Denver-Boulder-Longmont, Colorado metropolitan area. The KPCO institutional review board approved this study. KPCO has used a fully integrated EHR within KPCO since 1997; using KP HealthConnect EHR (KP HealthConnectTM KPHC, Epic Systems, Verona, Wisconsin) since 2004. The EHR includes all clinically relevant progress notes, telephone and email patient encounters along with an integrated computer provider order entry (CPOE) for all laboratory tests, imaging tests, medications and referrals. When a specialty department receives an electronic request for a traditional consultation, a staff member contacts the patient or the patient contacts the department for scheduling an appointment.

Clinicians may, in the course of their normal workflow within the EHR, use the CPOE function to submit an “advice only” consultation, for questions about etiology of a condition, diagnostic evaluation, treatment recommendations, or other questions about a particular patient. A specialist in the department reviews the advice request, which is attached to the patient’s electronic chart, facilitating easy review of the patient’s medical information. The specialist enters their recommendations into the consult request and returns the message to the requesting clinician’s electronic in-basket for review, usually within 24 hours.

During the study period, June 1, 2008 to Nov 22, 2008, we identified all adult (≥ 18 years of age) patient encounters in the EHR database which contained a VC or a TC to a specialty care department excluding requests for behavioral health consultation to maintain strict patient confidentiality. Self-referrals, durable medical equipment orders, and radiology orders were also excluded as these are not part of the advice messaging system. We reviewed VC and TC requesting patterns and only retained referral departments with evidence in the EHR of both types of consults (N = 33,390).

We randomly selected 270 VC and matched them to 270 similar TC that occurred in the same week. Consults were matched by patient age (±10 years), patient gender, specialty of consultation and the reason (3 digit ICD-9 code) for the consult. Selection and matching occurred every two weeks and selected patients were invited to participate in a telephone survey. Patients were contacted by phone within 2–4 weeks after the visit that contained the consultation request, and after obtaining informed consent (Additional file 1); we administered a survey assessing satisfaction with the clinical encounter and follow-up care related to the visit that included the consultation request. We adapted the survey from the modified Adult Ambulatory Care Consumer Assessment of Health Plans Survey (A-CAHPS) [32]. Survey questions (Additional file 2) assessed the specific visit in which the consultation was requested.

The referring physician for each selected TC and VC were invited by email to participate in an on-line survey (Additional file 3) about reasons for consultation (using a checklist based on previous pilot work [22] and modified from Ely’s taxonomy of clinical questions) [33], answers received, if answers met immediate patient care needs, likely or actual use of the information in subsequent patient care, impact of the consult process on physician work flow, and overall satisfaction with the consult. To avoid burdening physicians, a maximum of one survey per physician was sent each month, eliminating 115 consultations. Four consultations came from physicians who we were unavailable to survey. The final number of surveys sent to physicians was 421 (211 TC and 210 VC).

### Statistical methods

We calculated descriptive statistics and two-group comparisons between VCs and TCs. Although matching was used to select comparable VCs and TCs, survey selection as described above and physician non-response limited the number of matched pairs in the final survey sample, so that matched analytic methods were not used. Unmatched analyses of studies that match on exposure, rather than outcome, are generally less problematic since results will be accurate unless differential loss to follow-up introduces distortions. [34] The matching effort was successful in producing frequency matched comparison groups that did not differ on age or gender and restricted the sample to consult reason codes that were used for both TCs and VCs during the study time period. Differences between responding and non-responding physicians in use of TCs and VCs were evaluated using t-tests for mean consultation rates, Wilcoxon rank-sum tests for median consult rates, and Poisson regression models for ratios of VC to TCs. For variables measured at the patient level, p-values for differences between VCs and TCs were estimated using generalized linear mixed models that included a random effect for physicians (linear and logistic models estimated using SAS MIXED and GLIMMIX). SAS version 9.2 (Cary, N.C.) was used for all analyses.

## Results

### Comparison of virtual to traditional consults

**Figure 1:**
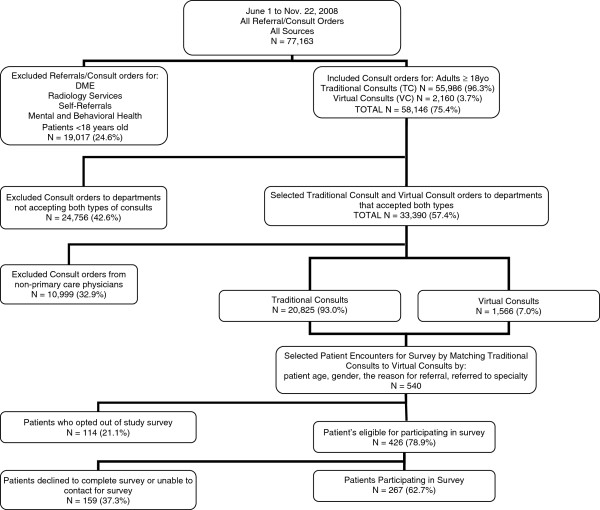
Flow diagram of the study.

We identified 77,163 referrals for consultation orders of all types originating from all sources (see Figure 1). The majority (N = 58,146, 75.4%) were for either VC or TC consultations for 44,034 unique adult patients. Physicians initiated the majority (47,144/58,146, 81.1%) of consultation requests. We focused our analysis on internal medicine and family medicine physicians, since they are the primary health care providers who coordinate care for their panel of patients and the primary users of the specialist’s advice. These physicians submitted 22,391 consultation requests for 19,441 unique adult patients; most of these were for TCs (20,825/22,391, 93.0%). Both types of consult requests occurred primarily for female patients (13,317/22,391, 59.5%).

The most common specialty departments for TCs were Physical Therapy (5,871/20,825, 28.2%), Orthopedics (2,641/20,285, 12.7%), Cardiology (2,129/20,825, 10.2%), Dermatology (2,079/20,285, 10.0%) and General Surgery (1,841/20,285, 8.8%). The most common specialty departments for VCs included Dermatology (282/1,566, 18.0%), Neurosurgery (211/1,566, 13.5%), Endocrinology (170/1,566, 11.0%), Neurology (124/1,566, 7.9%), and Orthopedic Surgery (96/1,566, 6.1%). Conditions that prompted the need for a consult were present for greater than 3 months in 76% of patients (203 of 267 patient survey respondents, 94.2% of the 97/103 VC and 75.1% of the 106/140 TC). One hundred fifty-one (56.6%) of survey respondent patients had already seen a specialist for the index condition in the last 12 months prior to the VC or TC (74/137, 54.0% with VC and 77/140, 55.0% with TC). Only 59.2% (160/240) of the VCs required conversion to a “face-to-face” specialist visit after the initial VC. This means that about 40% of physician questions could be handled through the virtual consultation method.

### Patient satisfaction survey

Some patients pro-actively opted out of participating in the survey (N = 114, 21.1%), some declined to complete the survey once they were called (N = 43, 8.0%), and we were unable to contact others (N = 115, 21.3%). The remaining 267 patients completed the survey, for a 49.4% overall response rate. Response rates were similar among patients who had VCs (N = 128; 47.9%) compared to TCs (N = 139; 52.1%; p = 0.30).

Patients who completed the survey were satisfied with the care they received (mean ratings greater than 8.5 on a 10 point Likert scale) from primary care and specialist physicians, irrespective of the kind of consult (see Table 1).

**Table 1:** Patient survey results of rating of quality of medical care (10 point Likert Scale)

						**Comparisons of VC vs TC**
	**N**	**Mean**	**Median**	**Min**	**Max**	p value*
**Rating of Primary Care Physician**						
**Virtual consult (VC)**	127	8.69	9	0	10	0.99
**Traditional consult (TC)**	138	8.69	9	4	10	
**Rating of Medical care**						
**Virtual consult (VC)**	128	8.67	9	0	10	0.51
**Traditional consult (TC)**	139	8.55	9	3	10	
**Rating of Specialist Recommendations**						
**Virtual consult (VC)**	41	8.71	10	0	10	0.63
**Traditional consult (TC)**	65	8.51	9	1	10	

* p values linear models with a random effect to adjust for patient clustering within physicians.

Total patient survey respondents, N = 267, however not all respondents answered all the questions so respondent numbers vary depending on the survey question.

### Physician survey

**Figure 2:**
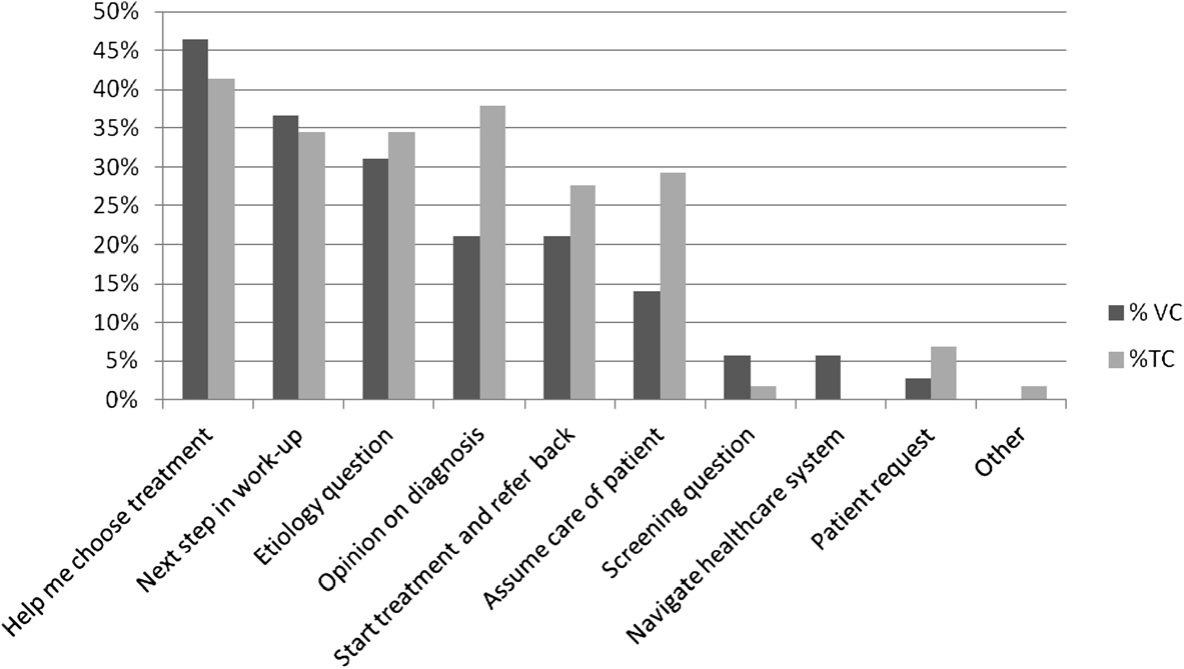
**Reasons given by requesting physicians for virtual and traditional consultation.** VC, Virtual consult; TC, Traditional consult. * p <0.05.

Of the 205 physicians surveyed, 88 (42.9%) replied to at least one survey, with no statistically significant differences between responding and non-responding physicians in the number of TCs and VCs ordered during the study period (Table 2). One hundred twenty-eight of 421 surveys (30.4%) were returned. Fifty-nine physicians completed one survey, 22 completed two surveys, three completed three surveys, and four completed four surveys. Of surveyed family physicians, 51.6% responded to at least one survey, compared with 36.5% of general internal medicine physicians (p = 0.03). Response rates on VCs (71/210; 33.8%) and TCs (57/211; 27.0%) were comparable (p = 0.13). Patients represented in the physician survey responses were mostly female (60.9%; p = 0.62 compared with survey non-responses), with a mean age of 53.7 ±11.8 years (p = 0.88 compared with survey non-responses). Patients receiving TCs or VCs in the returned survey sample did not differ by age or gender. A majority of physicians (57.8% for VC and 42.1% for TC) who responded to the survey indicated one reason for placing the consultation (range 1–5 reasons). Figure 2 shows that the reasons for TC and VC were generally similar, with more TCs than VCs intended to transfer care of the patient (p = 0.03), assist with diagnosis (p = 0.04) or start treatment and refer back to primary care (p =0.04). There were no differences between family and internal medicine physicians in reasons given for VC compared with TC (data not shown).

**Table 2:** Frequency of virtual and traditional consults among surveyed physicians

	**Responding PCPs n = 88**	**Non-responding PCPs n = 117**	**Responding vs non-responding PCPs p-value**
Virtual Consults (VC)			
Range	0-36	0-27	
Mean (SD)	7.4 (6.6)	6.8 (5.9)	0.46^*^
Median	5	5	0.56^†^
Traditional Consults (TC)			
Range	5-203	12-231	
Mean (SD)	86.6 (42.1)	92.5 (40.8)	0.31^*^
Median	87	89	0.47^†^
VC/TC (%)	11.4%	8.0%	0.19^‡^

^*^p value from *t*-test.

^†^ p value from Wilcoxon rank sum test.

^‡^ p value from poisson regression model for VC counts outcome, with log TC counts as the offset variable.

Model adjusted for over dispersion using scale parameter estimated by the square root of Pearson’s chi-square/degrees of freedom.

421 physician surveys sent to 214 physicians who ordered a TC and 207 to physicians who ordered a VC.

Many aspects of initiating VCs and TCs were similar. Few physicians (7.1%) called the specialty department before requesting a consultation. Only 2.4% of respondents indicated that the consultation process disrupted their workflow, with no difference between VCs and TCs. Physicians did not generally solicit patient preference for type of consultation, but more did so before TCs (41.2%) than VCs (26.5%), a difference that nearly reached significance (p = 0.15). Physicians reported that only 22.5% of patients volunteered a preference for one type of consultation, with no differences between TCs and VCs (Table 3). Within 3 weeks of the consultation request, 72.1% of respondents reported receiving information from the VC, compared with 33.9% of the TCs (p = <0.001), (Table 3). Requesting physicians did not differ in their satisfaction with the information provided or perception of the utility of TCs compared with VCs.

**Table 3:** Referring physician’s comments on the consultation requesting process, timeliness, utility, and satisfaction of information received from consultations

**Survey question**	**All consultations number/respondents (%)**	**VC number/respondents (%) 71 total responses possible**	**TC number/respondents (%) 58 total responsespossible**	**P -value for VC vs. TC**	**number of missing or cannot recall responses (n = 128 maximum)**
Referring physician called department before making consultation?	9/127 (7.1%)	4/70 (5.7%)	5/57 (8.8%)	0.52	1
Process of making consultation majorly or fairly disruptive to workflow?	3/123 (2.4%)	2/67 (3.0%)	1/56 (1.8%)	0.67	5
Referring physician solicited patient preference for type of consultation.	41/123 (33.3%)	18/68 (26.5%)	23/55 (41.2%)	0.15	5
Patients who expressed a preference for consultation type.	25/111 (22.5%)	16/60 (26.7)	9/51 (17.6)	0.38	17
Information received from consultant by the time referring physician completed the survey (2–3 weeks after consultation request)	68/124 (54.8%)	49/68 (72.1%)	19/56 (33.9%)	<0.001	4
Of referring physicians (n = 68) who received consultant information at the time of the survey, usefulness with information from consultation					
Useful (8–10 on 10 point Likert scale)	25/33 (75.6%)	17/21 (81.0%)	8/12 (66.7%)=	0.66	35
Somewhat useful (6–7 on 10 point Likert scale)	4/33 (12.1%)	2/21 (9.5%)	2/12 (16.7%)		
Not useful (1–5 on 10 point Likert scale)	4/33 (12.1%)	2/21 (9.5%)	2/12 (16.7%)		
Of referring physicians (n = 68) who received consultant information at the time of the survey, satisfaction with information from consultation					
Satisfied (8–10 on 10 point Likert scale)	26/36 (72.2%)	18/24 (75.0%)	8/12 (66.7%)	0.40	32
Somewhat satisfied (6–7 on 10 point Likert scale)	5/36 (13.9%)	2/24 (8.3%)	3/12 (25.0%)		
Not satisfied (1–5 on 10 point Likert scale)	5/36 (13.9%)	4/24 (16.7%)	1/12 (8.3%)		

*p values from logistic models with a random effect to adjust for clustering of patients within physicians.

Sixty-eight (53.1%) of 128 physicians surveyed provided answers to the question on the use of the information provided (this question could not be answered by physicians who had not yet received consultation information) (Table 4). Three-quarters of respondents indicated that all of the information provided was used in the care of the index patient; 7.4% reported using none of the information (TC vs. VC p = 0.75). Sixteen (23.5%) stated that they used index consultation information in the care of subsequent patients (TC vs. VC p = 0.78). Of the remaining 52 physicians, 18 indicated that they were at least somewhat likely (6 or higher on a 10 point Likert scale) to use the information from the index consultation in subsequent patient care (21 of the 52 physicians did not answer this question). There were no statistically significant differences (by specialty or number of surveys sent) between physicians who rated usefulness of or satisfaction with information from the consultation compared with those who did not answer these questions (data not shown).

**Table 4:** Use of information from consultation in care of index and subsequent patients

**Survey question**	**All consultations number/respondents (%)**	**VC number/respondents (%)**	**TC number/respondents (%)**	P value* VC vs. TC
Use of information from the specialist to care for the index patient				
All	51/68 (75.0%)	38/49 (77.6%)	13/19 (68.4%)	0.75
Some	12/68 (17.7%)	8/49 (16.3%)	4/19 (21.1%)	
None	5/68 (7.4%)	3/49 (6.1%)	2/19 (10.5%)	
Already used information from index consultation in care of other patients?	16/68 (23.5%)	12/49 (24.5%)	4/19 (21.1%)	0.78
If no, likely to use information in the future?				
Somewhat – very likely (6–10 on 10 point Likert scale)	18/52 (34.6%)	14/37 (37.8%)	4/15 (26.7%)	0.15
Not very – not likely (1–5 on 10 point Likert scale)	13/52 (25.0%)	6/37 (18.9%)	7/15 (46.7%)	
Did not answer question	21/52 (40.4%)	17/37 (43.2%)	5/15 (33.	

*p values from logistic models with a random effect to adjust for clustering of patients within physicians.

421 physician surveys sent to 214 physicians who ordered a TC and 207 to physicians who ordered a VC.

## Discussion

Little is known about the use of virtual consultations in an electronic health record. Our study demonstrated that using virtual consults via secure messaging within an EHR did not adversely affect patient’s perceived satisfaction with their care. While less than 7% of all the consults were virtual during the study time, when a provider opted to use a virtual consult to ask a question regarding patient care, 40% of the time a face-to-face consultation with the specialist was not needed. As providers (both primary care and specialists) and our patients gain more experience with this type of consultation, if the rate of virtual consults increases, we may experience improved access to specialists for patients who do require a face-to-face consultation. Patient satisfaction with care may increase if their questions are addressed by specialists without the burden of additional office visits. We do not know the optimal proportion between VC and TCs. With increasing experience, providers may learn which conditions do not need TCs and can be addressed with VCs. By incorporating this knowledge into caring for their other patients, they may improve the efficiency and quality of care they provide for their entire patient population.

The vast majority of study physicians relied on TCs, perhaps out of habit, comfort with traditional means of consultation, or slow adaptation of a new workflow. They seemed to somewhat selectively apply VCs, specifically by opting for TCs for confirmation of diagnosis, initiating a new treatment, or assuming care of the patient. We found that physicians received information from consultants more quickly using VCs than TCs. Otherwise, physicians utilized VCs and TCs for similar types of clinical questions and conditions. Most information from both TCs and VCs was used in care of index patients; many physicians had already or planned to use information from the consultation in the care of subsequent patients. TCs and VCs were minimally disruptive to physician workflow, and there were no differences in referring physician satisfaction between the two types of consultations. Most physicians did not determine patient preference for consult type, and physicians reported that most patients did not volunteer a preference for type of consultation.

Also, most patients did not express a preference for type of consult ordered, perhaps due to being unaware of the option of a VC (for those patients receiving traditional consultation), trusting their physician to determine the most appropriate type of consult, previous experience with consultation, lack of incentive to choose one form of consult over another, or truly having no preference.

Our results suggest that information from consultations is often applied not only in the care of the index patient, but also in caring for subsequent patients. Future application of new knowledge learned from a patient encounter is a desirable outcome of “point of care” learning; helping physicians document, process, and reflect on knowledge learned through practice-based consultation is a potential area for future outcomes-focused continuing medical education (CME) efforts [35]. Planning to use this information in the care of subsequent patients can be considered a type of commitment to change statement; these statements have been shown to be a reasonable predictor of future practice change [36-38].

### Limitations

Our study was conducted in a large group model practice with experience using a shared EHR, where all physicians are paid on salary, not on a relative value unit production system. This is an ideal setting for demonstrating how virtual peer-to-peer consultations using a secure electronic messaging system can potentially create bridges between often siloed care settings. Systems changes will be necessary in different healthcare environments for VCs to realistically occur. For example, in systems without integrated EHRs or in academic health centers providing consultation for rural or distant primary care physicians, mechanisms for efficient sharing of medical records (including HIPAA compliance) need to be developed. In systems where consultants are paid on a fee-for-service or production model, incentives would need to be provided for consultants to ‘accept’ virtual consults. However, as EHRs are slowly becoming more common, and data-sharing standards between different EHRs are being developed, we feel that our preliminary findings can inform care integration efforts in different care settings.

Both patient and physician survey respondents are subject to recall bias. Therefore we surveyed patients soon after the visit that prompted the consultation order (within 2–4 weeks) and we attempted to partially mitigate this bias among physicians by providing information about the patient so they could reference their chart notes about the visit prompting the consultation request. Patient and physician participation in the surveys was voluntary; therefore, survey respondents may represent a biased sample. We were unable to adjust for differential severity of disease state in patients since to gain sufficient patient numbers we matched on the reason for the consultation using just the first three digits of the ICD-9 code. Therefore, patients with more severe conditions or with multiple co-morbidities may have had a greater need for TC rather than VC.

While we achieved only a modest 30% physician survey return rate, a more representative 43% of physicians returned at least one survey. However, our return rates are not inconsistent with other surveys of busy physicians where relatively little incentive (other than continuing medical education credit) was provided [39,40], and the lack of difference in VC and TC frequencies between responding and non-responding physicians suggests we did not encounter a significant degree of response bias. With a larger sample size (on soliciting patient preference for type of consultation) and a larger response rate (particularly on the application or planned application of knowledge questions) we might have seen additional differences between TCs and VCs. We were not able to capture information on subjective judgments physicians may have made about their patients preferences for referral type. Physician respondents may have been predisposed to rate the consultations highly in order to not rate another department or colleague poorly, and our sample size did not permit comparisons between different specialty departments.

## Conclusions

If additional studies confirm these findings, increased use of VCs may decrease the need for face-to-face specialty encounters without a decrease in the patient’s perception in the quality of care, patient or referring physician satisfaction, with a quicker delivery of useful information to physicians requesting consultation. Using VCs for non-urgent consultation requests could potentially improve access to specialty care visits for patients with more urgent problems.

We believe the results of this investigation provide information useful for other specialties and health systems seeking to design virtual consultations to help streamline patient care. The results show the potential for using novel methods of health information technologies to explore new workflows to improve the affordability of health care, provide decision support to clinicians at the point of care, and inform clinicians how to provide alternative forms of medical care. This study also supports the anecdotal findings of the eHealth Initiative demonstration project which concluded EHR communications improved the referral process between primary care and the specialists [31].

Our study is a beginning step in gaining understanding if novel care delivery models can lead to “better, faster, cheaper” processes of care. In future studies we will need to perform a cost/benefit analysis between traditional and virtual consults to evaluate if virtual consults may lead to improved processes of healthcare at lower costs. If the value of the information a physician obtains via a virtual consult is equivalent to that obtained by a traditional consult, but results in saving the patient time (and potentially cost), then it may benefit both the patient and the physician. Therefore a virtual consult may be another way to potentially and conveniently meet patient needs and give physicians another tool for improving their practices.

In future work we plan to include additional incentives to increase percent of surveys returned, examine reasons physicians choose virtual consults for individual patients, assess the consistency of consultation advice with evidence, explore the applicability of our findings to other integrated health care systems as well as systems without electronic medical records or with lesser degrees of EHR implementation, and examine the patient outcomes of virtual consultations.

## Abbreviations

A-CAHPS: Adult ambulatory care consumer assessment of health plans survey; CME: Continuing medical education; CPOE: Computer provider order entry; EHR: Electronic health record; HIPAA: Health insurance portability and accountability act; ICD: International classification of diseases; KPCO: Kaiser Permanente Colorado; KPHC: Kaiser Permanente Health Connect; PCP: Primary care physician; TC: Traditional consults; VC: Virtual consults.

## Competing interests

TEP and DP are employees of the Colorado Permanente Medical Group. SS and KBW are employees of Kaiser Health Plan Colorado.

## Authors' contributions

TEP carried out the design of the study, assisted in the analysis, and drafted the manuscript. DP participated in the design, assisted in the analysis, and assisted in drafting the manuscript. SS performed all statistical analyses and participated with editing the manuscript. KBW performed the patient and physician surveys and collected data along with editing the manuscript. All authors read and approved the final manuscript.

## Authors' information

TEP is the physician manager for clinical reporting for the Colorado Permanente Medical Group (Denver CO) and a clinician researcher in the Institute for Health Research. He is the chair of the committee for integrating new technology with benefits and operations for Kaiser Permanente Colorado. Dr. Palen’s research focus is on the use of health information technology for enhancing models of care delivery.

DP is Director of Medical Education for the Colorado Permanente Medical Group (Denver CO) and the Permanente Federation (Oakland CA). He is also co-Director of the Kaiser Permanente Colorado Institute for Health Research's Center for Health Education, Dissemination and Research, and a member of the Board of Directors of the Accreditation Council on Continuing Medical Education. Among Dr. Price's areas of focus are examination of methods of effective physician learning, newer methods of learning for health professionals, and improving the translation of continuing education learnings into practice.

## Pre-publication history

The pre-publication history for this paper can be accessed here:

http://www.biomedcentral.com/1472-6947/12/65/prepub

## Supplementary Material

Additional file 1 Telephone script for introduction and verbal consent for virtual consults patient satisfaction survey. Click here for file

Additional file 2 Patient satisfaction survey. Click here for file

Additional file 3 Referring physician Survey. Click here for file
