# Supplementary material for: Comparing virtual consults to traditional consults using an electronic health record: an observational case–control study
Source: BMC Med Inform Decis Mak. 2012 Jul 8;12:65. doi: 10.1186/1472-6947-12-65 (PMC3502437; doi:10.1186/1472-6947-12-65)
Supplement: Additional file 1 — Telephone script for introduction and verbal consent for virtual consults patient satisfaction survey. [file 1472-6947-12-65-S1.doc]

### Additional file 1 – Telephone Script for Introduction and Verbal Consent for Virtual Consults Patient Satisfaction Survey

# **IDENTIFICATION**

**Hello, my name is (___). I’m calling on behalf of Kaiser Permanente Colorado at the Institute for Health Research.**

**May I please speak with ________________________________?**

(No, s/he isn’t home.)

**Could you tell me the best time to contact _____________________________?**

**If it’s more convenient for (_______) to call me back, I can be reached at (xxx-xxx-xxxx). If I am not available they may leave a message on my 24-hour Voice Mail and leave their name, phone number and a date and time that would be best to reach them.**

**Thank you for your time.**

(No, s/he wouldn’t be able to answer your questions because of his/her health condition.)

**Thank you for your time.**

(No, we’re not interested.)

**Thank you for your time.**

**(If non-participant asks for additional information about the study:** **“It’s our policy not to give out further information but I would be happy to explain it to (_______).”)**

**Voicemail Script:**

**Hello, my name is (___) from Kaiser Permanente Colorado at Institute for Health Research. I am calling (_______) regarding a letter he/she received from us about a week ago. So I know a better time to speak with you, please call my 24-hour Voice Mail at (xxx-xxx-xxxx) and leave your name, phone number and a date and time that would best to reach you. Thank you.**

**LETTER VERIFICATION**

**I’m calling to tell you about a research study we are conducting on patient satisfaction. Do you remember receiving our letter about this study in the mail?**

*(If yes)* **Great. We’d like to ask you some questions about your satisfaction with a recent primary care appointment. Just to remind you…..** *(Continue to Introduction)*

*(If no)* **The letter invites you to participate in this study by Kaiser Permanente Colorado and includes information about your rights as a participant in this study, so we’d like to make sure you are informed before continuing with the interview. Before I resend the letter, would you be interested in learning more about this study?**

*(If no)* **Thank you very much for your time.**

*(If yes)* **We want to make sure** **the mailing address we have on file is correct. Can you verify your current address?**

*(If yes)* **Great! I’ll resend that letter out to you. Would you like to hear more about the study at this time or wait until you receive the letter?**

*(Hear more)* **Great, so let me tell you about our study...** *(Continue to Introduction)*

*(Wait)* **Okay, I’ll resend that letter out and we’ll try calling you back in a few days. Thank you.** *(End call, resend letter)*

*(If no)* **I’ll make a note of the correct address.** *(Make note on call log and in database)* **Great! I’ll resend that letter out to you. Would you like to hear more about the study at this time or wait until you receive the letter?**

*(Hear more)* **Great, so let me tell you about our study...** *(Continue to Introduction)*

*(Wait)* **Okay, I’ll resend that letter out and we’ll try calling you back in a few days. Thank you.** *(End call, resend letter)*

**INTRODUCTION**

**The purpose of this study is to evaluate a new secure e-mail type of communication method used by primary care providers to consult with specialists about patient care. This type of consultation takes place entirely within HealthConnect, our electronic medical record.  As part of this study we would like to compare patients’ experience when this new method was used and compare it to patients’ experience where a traditional referral to a specialist was used. This research study is designed to be a one-time survey conducted over the phone. You will be asked questions about your satisfaction with a recent primary care appointment you had where either the new communication method or a traditional referral was used. The information obtained from this study will be important in helping Kaiser Permanente better understand the potential benefits and challenges for this new communication method.**

**Is this a good time to talk?**

[ ] Yes *Continue to Consent*

[ ] No **When would be a good time to talk?** *(Record time on call log)*

**Thank you for your time.**

**CONSENT**

This study involves a one time telephone interview that will take about 10 minutes of your time. Your participation in the study is completely voluntary. Your decision whether or not to participate in the study will not affect your medical care. If you decide to participate, you are free to change your mind and discontinue participation at any time without any effect on your medical care or eligibility for future care or membership in Kaiser Foundation Health Plan of Colorado.

You don’t have to answer any questions that make you uncomfortable, and you may stop the interview at any time. Included with the letter you received was an information sheet that tells you about the study, your rights, and gives the name and telephone number of the person to contact if you have questions or change your mind about participating in the study.

I want to make sure you understand what personal health information we will be collecting and how this will be kept private. All of your answers will be kept confidential. The only people who will have access to your answers will be the study investigators. The information will not go to your doctor or health plan or into your medical record. The information will be stored in secure, locked cabinets and will be destroyed after the completion of the project or after the requisite six years for PHI tracking, whichever is latest. You will not be compensated for the study and there are no foreseeable benefits for you in participating. Do you have any questions regarding what I have just discussed with you?

I want to make sure you have the names and numbers of people you can contact if you have any questions after our discussion today. If you have any questions or concerns about your rights as a participant in this study, you can call the Kaiser Permanente Colorado Institutional Review Board at 303- 614 -1309. If you have any questions or concerns about the study, you can contact the study Project Manager, Kristin Wallace, at 303-614-1221. These names and telephone numbers are also on the letter we sent out to you.

**Would you like to participate in this study?**

**[ ] No** Thank you very much for your time.

[ ] Yes **Proceed to patient satisfaction survey**
