# Supplementary material for: Comparing virtual consults to traditional consults using an electronic health record: an observational case–control study
Source: BMC Med Inform Decis Mak. 2012 Jul 8;12:65. doi: 10.1186/1472-6947-12-65 (PMC3502437; doi:10.1186/1472-6947-12-65)
Supplement: Additional file 2 — Patient satisfaction survey. [file 1472-6947-12-65-S2.doc]

**Additional file 2 – Patient satisfaction survey**

(NOTE: The parenthetical numbered items refer to the actual A-CAHPS survey questions, A-CAHPS question #31, What is your age? and A-CAHPS question #32, Are you male of female?, were obtained from administrative data.)

I’m going to ask you about an appointment you had with Dr. ______ on <date> regarding <condition/complaint>. *(This appointment may or may not have been an in person visit)*

1. Is this doctor your personal care physician? Y N
2. (A-CAHPS question #4) In the last 12 months, how many times did you visit this doctor to get care for yourself? ________
3. (A-CAHPS question #14) Did this doctor explain things in a way that was easy to understand? Y N
4. (A-CAHPS question #20) Did this doctor spend enough time with you? Y N
5. (A-CAHPS question #23) Using any number from 0 to 10, where 0 is the worst doctor possible and 10 is the best doctor possible, what number would you use to rate this doctor?
6. (A-CAHPS question #RC1) Would you recommend this doctor to your family and friends? Y N
7. (A-CAHPS question #RV10) Using any number from 0 to 10, where 0 is the worst medical care possible and 10 is the best medical care possible, what number would you use to rate the medical care you received during this visit with this doctor? __________
8. (A-CAHPS question #RV11) Please tell us how this doctor’s office could have improved the care and services you received at this visit.
9. Did this doctor ask you if you had a preference between: you seeing a specialist in person, or this doctor seeking advice from a specialist and getting back to you?

Y N Don’t Remember

1. Please choose from the following statements that best reflect this visit:
   1. I was given a referral for a visit to see a specialist
   2. This doctor discussed getting information from a specialist with me and later contacted me to share the specialists’ recommendations
   3. This doctor discussed getting information from a specialist with me but was only going to contact me if changes to my care was needed
   4. This doctor discussed getting information from a specialist with me but I haven’t heard about recommendations at this time
   5. This doctor and I did not discuss getting additional specialist information at this visit

**(If answer a above, go to questions 11, 12 & 13 then skip to 16)**

**(If answer b above, skip to question 14 & continue)**

**(If answer c, d or e above, skip to question 16 & continue)**

1. Have you see seen the specialist yet? Y N

If no, skip to question 16.

1. Using any number from 0 to 10, how satisfied are you with the recommendations made by the specialist for this problem? __________
2. Did you have any questions about the recommendations made by the specialist for this problem? Y N
   1. If YES, using any number from 0 to 10, how satisfied are you with your ability to ask questions about the specialist recommendations for this problem? __________

Skip to question 16.

At this visit, the doctor you saw asked for input from a specialist to help take care of your concern at that visit. Please tell us:

1. Using any number from 0 to 10, how satisfied are you with the time it took you to hear about additional recommendations for care of this problem? __________
2. (A-CAHPS question #PK2) Did this doctor who asked the specialist for input seem informed and up-to-date about the care recommended by specialists for your problem? Y N
3. If this doctor is not your personal care physician, does your personal care physician seem informed and up-to-date about the care recommended by specialists for your problem?

Y N N/A (this doctor is my personal care physician) Don’t know

1. (A-CAHPS question #26) In general, how would you rate your overall health?

Excellent Very Good Good Fair Poor

1. (A-CAHPS question #27) In the past 12 months, have you seen a doctor or other health provider 3 or more times for the same condition or problem that you had a referral for at this visit? Y N
2. (A-CAHPS question #SC5) How many specialists have you actually seen in the last 12 months for this condition?
3. (A-CAHPS question #28) Is this a condition or problem that has lasted for at least 3 months? (*Do not include pregnancy or menopause*). Y N
4. (A-CAHPS question #33) What is the highest grade or level of school that you have completed?

Less than HS HS Grad Some College College Grad Post College Grad

1. (A-CAHPS question #35) With which of the following racial/ethnic group(s) do you identify? You may choose more than one.
   1. American Indian/ Alaska Native
   2. Asian
   3. Native Hawaiian or other Pacific Islander
   4. Black or African American
   5. White
   6. Hispanic or Latino(a)
   7. Other ______________________
   8. Decline
