# Supplementary material for: Comparing virtual consults to traditional consults using an electronic health record: an observational case–control study
Source: BMC Med Inform Decis Mak. 2012 Jul 8;12:65. doi: 10.1186/1472-6947-12-65 (PMC3502437; doi:10.1186/1472-6947-12-65)
Supplement: Additional file 3 — Referring physician Survey. [file 1472-6947-12-65-S3.doc]

### Additional file 3 – Referring Physician Survey

1. Issue(s) that prompted this referral (choose all that apply):

- question about etiology of condition
- question about screening
- question about what to do next to evaluate the patient
- requested consultant's opinion on what he/she thinks is the diagnosis for this patient
- requested assistance in selecting most appropriate treatment
- requested specialist to initiate treatment and have patient follow-up with me
- requested specialist to assume care for this patient for this condition
- question related to figuring out how to navigate the Kaiser system and/or coordinate care
- other:

2. Before making a referral for this patient, did you **also** make a phone call to the referral department?

- Yes
- No
- Don’t remember

3. Did you ask the patient if they preferred to see a specialist in-person for this condition?

- Yes
- No
- Don’t remember

4. Did the patient express an interest for having a traditional or KPHC advice referral?

- Yes
- No
- Don’t remember

5. What specific questions did you have for the consultant?:

1.

2.

3.

6. What were the answers to your questions?:

1.

2.

3.

7. Did you use the information in the consult to provide subsequent care to this patient?

- all the information
- some of the information (please specify which information you used):
- none of the information
- I haven’t yet heard back from specialist (if this is selected, skip to question #9 and continue)

8. On a 10 point scale (10 = exceedingly well, 1 = not at all), how well did the information provided in the consult meet your needs in caring for this patient?:

9. How disruptive to your work flow was the process of making the referral for this patient?

- Not at all disruptive
- Minimally disruptive
- Fairly disruptive
- Majorly disruptive

10. On a 10 point scale (10 = highly satisfied, 1 = highly dissatisfied), satisfied were you overall with this consultation?

11. Have you used any of the information from this consult in the care of subsequent patients?

- no

If not, on a 10 point scale, how likely are you to use this information in future patient care?

If not likely (5 or less), what barriers do you anticipate might keep you from using this information?

- yes

If yes, please specify which information:

- not applicable, I haven’t yet heard back from the specialist
